# Deep sequencing and expression of microRNAs from early honeybee (*Apis mellifera*) embryos reveals a role in regulating early embryonic patterning

**DOI:** 10.1186/1471-2148-12-211

**Published:** 2012-11-02

**Authors:** Lisa Zondag, Peter K Dearden, Megan J Wilson

**Affiliations:** 1 Laboratory for Evolution and Development, Genetics Otago and the National Research Centre for Growth and Development, Department of Biochemistry, University of Otago, P.O. Box 56, Dunedin, 9054, New Zealand; 2 Developmental Biology Laboratory, Department of Anatomy, University of Otago, P.O. Box 56, Dunedin, 9054, New Zealand

## Abstract

**Background:**

Recent evidence supports the proposal that the observed diversity of animal body plans has been produced through alterations to the complexity of the regulatory genome rather than increases in the protein-coding content of a genome. One significant form of gene regulation is the contribution made by the non-coding content of the genome. Non-coding RNAs play roles in embryonic development of animals and these functions might be expected to evolve rapidly. Using next-generation sequencing and *in situ* hybridization, we have examined the miRNA content of early honeybee embryos.

**Results:**

Through small RNA sequencing we found that 28% of known miRNAs are expressed in the early embryo. We also identified developmentally expressed microRNAs that are unique to the Apoidea clade. Examination of expression patterns implied these miRNAs have roles in patterning the anterior-posterior and dorso-ventral axes as well as the extraembryonic membranes. Knockdown of Dicer, a key component of miRNA processing, confirmed that miRNAs are likely to have a role in patterning these tissues.

**Conclusions:**

Examination of the expression patterns of novel miRNAs, some unique to the *Apis* group, indicated that they are likely to play a role in early honeybee development. Known miRNAs that are deeply conserved in animal phyla display differences in expression pattern between honeybee and *Drosophila*, particularly at early stages of development. This may indicate miRNAs play a rapidly evolving role in regulating developmental pathways, most likely through changes to the way their expression is regulated.

## Background

A major component of the transcriptome of animals consists of non-protein coding RNAs [1-3]. Micro-RNAs (miRNAs) are a subset of small non-coding RNAs that are 18-24 nucleotides long and have a key role in regulating gene expression in eukaryotes. They are produced from a primary full-length transcript (pri-miRNA), which is cleaved to form hairpin structures around 70 nucleotides in length. These are called precursor miRNAs (pre-miRNAs) and are exported to the cytoplasm to be processed further to functional mature miRNAs by the ribonuclease Dicer [4,5]. Once assembled into the RNA-induced silencing complex (RISC), the miRNA acts on its target by binding to complementary sequences present in the 3^′^ untranslated regions (UTR) of the target mRNA [6]. This results in either translational repression or mRNA cleavage, thus providing another level of gene regulation [7].

There is accruing evidence to suggest that miRNAs play a role in regulating multiple developmental pathways, including fundamental developmental processes of animal development such as axis formation and organ morphogeneis. Many miRNAs are expressed in developmentally restricted patterns [8,9], for example, examination of miRNA expression in *Drosophil*a during embryogenesis found that many are expressed in restricted patterns along the anterior-posterior and dorso-ventral axes and in specific tissues. Their expression is developmentally regulated, often by their own promoter and regulatory elements, similar to developmental protein coding factors [10]. Loss of miRNA function often results in defective development and patterning [11-14], indicating an essential role in animal development. MiRNAs are also proposed to provide developmental stability particularly under times of environment stress, acting to buffer developmental pathways [15].

Many miRNA families are ancient and can be traced to more basal animals. Expression analysis and functional studies indicate that their roles are often conserved [8]. As single miRNAs can bind to many different mRNAs to regulate their expression, they can potentially impact on several regulatory pathways [16]. Thus any changes to the way miRNAs are expressed are likely to impact multiple developmental processes. Many miRNAs have been found to be specific to particular phylogenetic groups, some found only in particular lineages. Over 40 miRNA families arose early in the lineage leading to the vertebrates, and it has been suggested that these contributed to the evolution of vertebrate complexity [17].

Here we have profiled the miRNAs expressed during early embryogenesis in the honeybee (*Apis mellifera*) to determine if they are likely to play a significant role in honeybee embryogenesis. The expression and function of miRNAs in insect development has to date only been investigated in *Tribolium* and *Drosophila*[8,18]. Like *Drosophila*, *Apis* development begins with a syncytial blastoderm stage prior to cellularisation, where much of the body patterning information is established [19]. Both *Apis* and *Drosophila* are considered long germ band insects where segmentation occurs across the whole body [20]. However there are some significant differences, notably in patterning of the extraembryonic membranes. In *Drosophila* the extraembryonic membranes are patterned as one tissue, the amino-serosa, a process regulated by the transcription factor zen [21]. In honeybee, these membranes are patterned separately, although both tissues still require zen [22]. There are also significant differences in the nature of the regulatory networks required to pattern the anterior-posterior axis [22-24]. The honeybee genome has been sequenced and 168 miRNAs have so far been predicted. The *Drosophila* genome encodes at least 430 miRNAs (mirBase). We examined the expression of honeybee miRNAs during early development, by deep sequencing and *in situ* hybridization. This included developmental stages at which the anterior-posterior and dorso-ventral axes have been established, patterning including segmentation is underway, just prior to gastrulation. As the pattern of miRNA expression is often reflective of their function in a developmental process or tissue patterning, we examined the expression of eight miRNAs identified in our study. Additionally, RNAi knockdown of *Dicer* during early embryogenesis indicated that small RNAs are likely to contribute to early honeybee embryo development.

## Results and discussion

### Abundance and expression of previously known miRNAs

**Figure 1:**
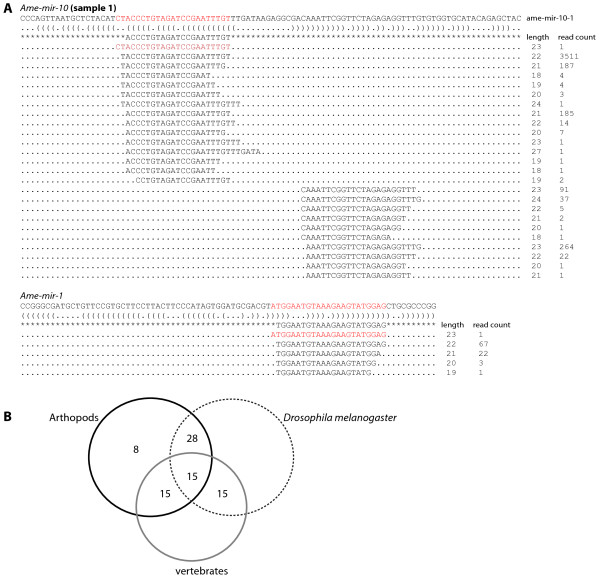
**Alignment of sequence reads back to the miRNA precursors for mir-184 and mir-1. **(**A**) An example of mapping sequence reads back to the pre-miRNA precursor. Bracket-notation for secondary structure is shown below the precursor and the mature miRNA sequence (miRBase) is flacked by asterisks. The number of reads giving rise to a particular sequence is noted beside each sequence. Highlighted in red is the most abundant small RNA read. (**B**). Venn diagram of the distribution of known miRNAs from the honeybee embryo small RNA libraries between *Drosophila*, other arthropods and vertebrates.

Total RNA was extracted from honeybee embryos aged from 24 to 30 hours old (stages 4 to 5). Approximately 20 million reads were generated per sample, corresponding to 3 million unique reads (Table 1). Almost 70% of unique small RNA tags were mapped back to the *Apis mellifera* genome (Table 1). The number of reads for known (i.e. present in miRBase) *Apis mellifera* miRNAs are shown in Table 2 and an example of an alignment is shown in Figure 1A. This provides further experimental evidence that these are transcribed miRNAs in the honeybee genome. Of the 168 mature miRNA in miRBase, 45 were represented in both samples. This indicates that almost 28% of known miRNAs are expressed in the early honeybee embryo, implying a possible role for these in regulating developmental pathways. An example of read alignments to precursor miRNA are shown Figure 1A.

**Table 1:** Summary of small RNA sequencing results

	**Sample 1**	**Sample 2**
Total number of reads	20120856	21480569
Clean reads	19563073	20873296
Number of reads mapped back to genome	13200933	14892414
Number of reads map to annotated pre-miRNA	21450	18469

**Table 2:** Profile of known miRNAs in present in 24-30 hour embryos

**miRNA**	**Sample 1**	**Sample 2**
ame-miR-100	101	73
ame-miR-1	94	5
ame-miR-71	269	88
ame-miR-3759	5	107
ame-miR-184	5294	4431
ame-miR-927	21	22
ame-miR-275	22	15
ame-miR-7	158	73
ame-miR-279	23	8
ame-miR-8	9	8
ame-miR-92b	103	149
ame-miR-283	8	16
ame-miR-3756	362	264
ame-miR-2	167	261
ame-miR-263b	6	16
ame-miR-3785	13	14
ame-miR-11	15	26
ame-miR-3747a	606	143
ame-miR-279c	544	310
ame-miR-315	4	13
ame-miR-13a	10	3
ame-miR-12	8	20
ame-miR-2944	2452	1177
ame-miR-263	39	141
ame-miR-375	6	6
ame-miR-279b	335	318
ame-miR-34	9	6
ame-miR-750	8	7
ame-miR-989	8	4
ame-miR-9c	89	69
ame-miR-3477	211	199
ame-miR-3715	15	35
ame-miR-10	3923	3561
ame-miR-125	49	87
ame-miR-3747b	63	23
ame-miR-996	17	3
ame-miR-9a	2214	1977
ame-miR-92a	175	214
ame-let-7	135	185
ame-miR-3786	31	32
ame-miR-252	14	10
ame-bantam	13	6
ame-miR-317	360	354
ame-miR-306	608	619
ame-miR-3791	592	707

We determined if these miRNAs are also present in the genomes of other phyla. Of the miRNAs we isolated from honeybee embryos, 36 are also present in the genomes of other arthropods. 15 of these are present in the vertebrate lineage, indicating these are likely to be ancestral (Figure 1B). Less conservation was observed between *Apis* and *Drosophila* miRNA content which has also been noted previously between *Tribolium* and *Drosophila*[18], indicating that Arthropod groups (other than Diptera) have more miRNA content in common to each other than when compared separately to Diptera.

To determine if the developmental expression patterns of miRNAs with orthologues in both honeybee and *Drosophila* were conserved, we examined the expression of four miRNAs detected in our study, with expression and functional data of their orthologue in *Drosophila*.

*In situ* hybridisation was performed using RNA probes designed to bind to the pri-miRNA, the longer transcript from which the pre-miRNA is produced, prior to export from the nucleus. This strategy has been successfully used in previous studies illustrating that it reflects the mature miRNA expression (when detected using LNA probes) [25]. The probes used detect nascent transcripts before processing by Drosha RNase III enzyme and thus are expected to detect nuclear dots rather than the cytoplasmic staining produced with probes against an mRNA transcript (for example see Additional file 1: Figure S3).

**Figure 2:**
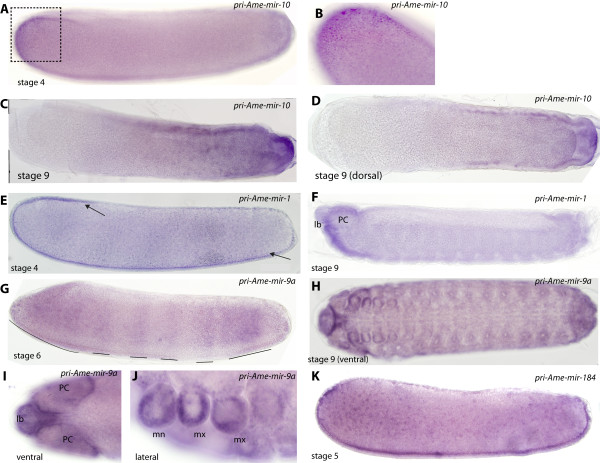
**Expression of honeybee pri-miRNAs present during embryogenesis. **Embryos are shown with anterior to the left and dorsal side up, unless stated otherwise. Scale bar is 100 μm. (**A**) Expression of *pri*-*Ame*-*mir*-*10* at stage 4 is detected in the ectoderm at the anterior pole of the embryo. (**B**) Higher magnification image showing nuclear staining in anterior ectodermal cells. (**C** and **D**) Expression of *pri*-*Ame*-*10* at stage 9 is found in the posterior mesoderm (ventral orientation in D) and in the posterior terminal segment. (**E**) *Pri*-*Ame*-*mir*-*1* is expressed central ventral ectoderm at stage 5. (**F**) By stage 9 *pri*-*Ame*-*mir*-*1* RNA is detected in regions of the procephalic lobes (PC), and within the labrum (lb). Expression is also detected in the posterior anal plates. (**G**) *Pri*-*Ame*-*mir*-*9a* is detected in broad bands of ectodermal cells across the anterior-posterior axis. Staining was absent from the dorsal and ventral regions of the stage 6 embryo. (**H**) Expression of *pri*-*Ame*-*mir*-*9a* is detected through the epithelium at stage 9. (**I**) Strong epithelial staining for *pri*-*Ame*-*mir*-*9a* in the labrum and procephalic lobes at stage 9. (**J**) *Pri*-*Ame*-*mir*-*9a* is also present in the epithelium of the head appendages, maxillae (mx) and mandibles (mn) but absent from the internal regions of these structures, where the sensory neurons are present. (**K**) Expression of *pri*-*Ame*-*mir*-*184* is detected through mesoderm from anterior, ventral and posterior of the embryo but absent from the ventral side of the stage 4 embryo.

Mir-10 is a widely conserved miRNA in both sequence and genomic location in both invertebrates and vertebrate *Hox* gene clusters. It is located within the *Hox* complex in *Drosophila* between the *deformed* (dfd) and *sexcombs reduced* (Scr) Hox genes and has been predicted to directly regulate mRNA translation of nearby *Hox* genes that contain mir-10 binding sites in their 3^′^UTRs [26]. However ectopic expression of *Dmemir10* had no significant effect on the expression of predicted *Hox* targets (Scr and Abd-B) [25], suggesting that they may not be biologically relevant targets for mir-10 in a laboratory setting. *Apis mellifera mir10* (*Amemir10*) is also located in the *Hox* gene complex between *Amdfd* and *AmScr* (Additional file 2: Figure S4). We detected expression of this miRNA during embryogenesis (Table 1 and Figure 1A). *In situ* hybridisation analysis revealed expression of *priAmemir10* at the anterior pole of the embryo (Figure 2A and B). Later *priAmemir10* RNA expression (stage 9; approximately 48 hours old) was detected throughout the posterior two-thirds of the embryo, in the posterior terminal segment and the underlying mesoderm (Figure 2D). This is consistent with expression of a *Hox* regulator, as it shows limited expression along the anterior-posterior axis. In comparison, *Drosophila priDmemir10* expressed in a broad band across the middle of blastoderm embryos and, following germ band extension, it is expressed in the posterior half of the embryo, in the anal pad, ventral neuroectoderm and hindgut [25]. This indicates that while there are similarities in late embryonic expression of *mir10* between *Drosophila* and honeybee embryos, *mir10* expression in early embryos is quite different. This may indicate a shift in *mir10* function in early embryogenesis between Diptera and hymenoptera.

Previous deep sequencing of *Drosophila* embryonic RNA revealed that most of the reads correspond to the 3^′^ arm of the mir-10 precursor [27], although RNA expression patterns of both mir-10-5p and mir-10-3p is similar in *Drosophila* embryos [25]. We found that the 90% of sequence reads are from the 5^′^ arm of the pre-miRNA in honeybee embryos, indicating that mir-10-5p is responsible for the majority of mir-10 function (Figure 1). This has also been found for mir-10 in *Tribolium*[18]. Changes to which part of the pre-miRNA strand provides the dominant or functional miRNA sequence (arm switching) is proposed to be one mechanism of miRNA evolution to drive miRNA diversification [28]. Our results and those from *Tribolium*[18] would indicate that the ancestral dominant arm was the mir-10-5p (producing the mature miRNA) and that this has switched during *Drosophila* evolution to the mir-10-3p arm. However, while only 10% of the total reads for pre-mir-10 were from the 3p arm in honeybee embryos (Figure 1), it was still a significant number (425) and more abundant that some of the other miRNAs detected (Table 2), indicating that the mature miRNA from this arm of the mir-10 hairpin (mir-10-3p) may have a distinct role during honeybee development.

Mir-1 is a highly conserved miRNA that has been suggested to play a role in myogenesis in *Drosophila* and vertebrates [29-31]. We detected low numbers of reads for *Amemir1* in both our samples (Table 1), but because of its conservation between vertebrates and invertebrates, we examined its RNA distribution. Staining for *priAmemir1* was weakly detected through ventral and anterior mesoderm of the embryo (Figure 2E). By stage 9, expression was found in the anterior of the embryo within the area of the labrum and in restricted regions of the procephalic lobes (Figure 2F). No expression was detected in mesoderm-derived tissues late in development. In *Drosophila*, *Dmemir1* is also expressed in ventral mesoderm [29,32] but continues to be expressed throughout mesodermal tissues later in development and is required for muscle and cardiac patterning [29,33]. This expression pattern of *Dmemir1* is regulated by Twist (Twi), a pro-mesoderm transcription factor [29]. Examination of the upstream and downstream regions surrounding the *Amemir1* coding region failed to find any significant cluster of Twi binding sites (Additional file 3: Figure S5), possibly explaining the lack of *Amemir1* expression in the *Apis* mesoderm. This implies loss of an enhancer element(s) for directed expression in ventral mesoderm in the honeybee, as mesodermal expression is also found in vertebrates suggesting it is a more ancient pattern for mir-1.

Mir-9a is a conserved microRNA in sequence but with differing functions in invertebrates and vertebrates. *Drosophila Dmemir9a* is expressed in the dorsal ectoderm and neuro-ectoderm at early stages [32] and ectodermal epithelial cells including the epithelial surfaces of the head appendages. *Dmemir9a* homozygous mutants survive to hatching and are fertile but produce ectopic sensory neurons, indicating a role in negatively regulating neuron number [34]. In vertebrates, however, while *mir*-9a is expressed in the developing brain it has a differing role, positively regulating neurogenesis [35,36]. Sequencing data indicated that *Ame-mir-9a* was expressed (Table 1) during honeybee early development. *In situ* hybridisation at stage 5, just prior to gastrulation, detected mir-9a throughout the head ectoderm, and then in broad ectodermal stripes across the middle of the embryo to the posterior terminus (Figure 2G). Expression was absent in the dorsal and ventral sides of the embryo (Figure 2G). By stage 9, *priAmemir9a* RNA was found throughout the epidermis but was weak or absent in neurons of the central nervous system (CNS) (Figure 2H). Expression was strongest in the epidermis of the procephalic lobes and labrum, and no staining was found within cephalic and labrum regions where the neuronal cells are present (Figure 2I). A similar pattern of expression was also found in the mandibles and maxillae, with all appendages exhibiting strong staining around epithelium for *priAmemir9a* but absent from the central regions of the appendages, where the sensory neuronal tissue are predicted to be (Figure 2J). Therefore *mir9a* RNA expression in both honeybee and *Drosophila* show similar patterns, with strong epithelial cell basis, consistent with a conserved role in regulating production of sensory organ neuronal cells and suppressing sensory neural fate in the surrounding epithelia. In vertebrates, mir-9a has a quite different role in positively regulating neurogenesis, indicating that both its expression and function has changed significantly in the vertebrate group or this developmental role of mir-9a is particular to the insect group.

Mir-184 is also conserved between invertebrates and vertebrates, and has been shown to play an important role in axis formation and oogenesis in *Drosophila*[37]. Ame-mir-184 had the highest read count of any miRNA in our small RNA library (Table 1). *PriAmemir184* RNA was detected in the mesodermal cells throughout the embryo except the dorsal side of the embryo where extraembryonic membranes differentiate (Figure 2K). Previous studies have shown that *Dmemir184* is expressed along the mesoderm on the ventral side of the embryo [32,37].

### Prediction and expression of novel miRNAs

Candidate miRNAs were identified in our small RNA libraries using the prediction software Mireap [38] that takes into consideration typical miRNA features, such as the presence of a Dicer cleavage site, and correct secondary structure. Sixteen previously unknown miRNAs satisfied these criteria, were present in both RNA samples (Table 3; Additional file 4), and were mapped back to the genome. To determine if these miRNAs were also present in other insects, we used BLAST searches with the mature microRNA sequence to hymenopteran genomes, and then examined the presence of a stem-loop structure in the predicted precursor for each significant hit to determine if they are likely to represent orthologous miRNAs. Eight novel Ame-miRNAs were also present in the *Bombus* (Bumble bee) genome and two within *Atta cephalotes* (Ant) genome but only one within the *Nasonia* (Jewel wasp) genome (Table 3). This indicates that many of these novel miRNAs may have arose after branching of the Apoidea group or have been lost from other lineages. We examined the expression patterns of four of these newly identified miRNAs during honeybee embryogenesis.

**Table 3:** Novel miRNA profile and presence (+) or absence (-) in the genomes of other insects

***Candidate***	**Reads**	**Reads**	***Bombus***	***Nasonia***	***Atta cephalotes***
***miRNA***	**Sample 1**	**Sample 2**			
*mir*-*0002*	197	144	+	-	-
*mir*-*0003*	106	82	+	-	-
*mir*-*0004*	7	8	+	-	-
*mir*-*0005*	1642	2263	+	+	+
*mir*-*0006*	30	35	-	-	-
*mir*-*0007*	5688	5036	+	-	-
*mir*-*0008*	59	29	+	-	+
*mir*-*0009*	6	9	+	-	-
*mir*-*0010*	44	30	-	-	-
*mir*-*0011*	6	14	-	-	-
*mir*-*0012*	2095	1152	-	-	-
*mir*-*0013*	10	17	-	-	-
*mir*-*0014*	405	206	-	-	-
*mir*-*0017*	405	206	-	-	-
*mir*-*0018*	37	10	-	-	-
*mir*-*0020*	87	88	+	-	-

**Figure 3:**
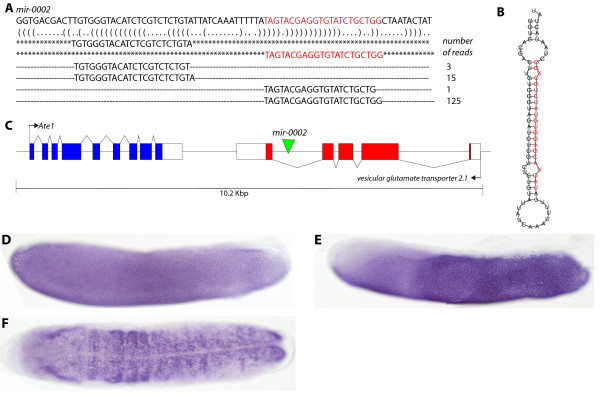
**Expression and sequence of novel Ame-mir-0002. **Embryos are shown anterior to the left, dorsal side up. (**A**) Sequence of the predicted mir-0002 precursor, aligned below are the sequence reads and associated read counts. Highlighted in red is the sequence with the highest read count. Bracket-notation of secondary structure is beneath the precursor sequence. (**B**) The predicted mir-0002 hairpin structure, with the most abundant read sequence highlighted in red. (**C**) Genomic location of the predicted *pre-mir-0002* miRNA is within the vesicular glutamate transporter gene on chromosome 11. (**D**) Expression of *pri*-*Ame*-*mir*-*0002* during embryogenesis. At 24 hours following oviposition (stage 4), expression of *pri*-*Ame*-*mir0002* is detected through the ectoderm. (**E**) By stage 6 (30 hours), staining for *pri*-*Ame*-*mir*-*0002* is lost from the regions of the ectoderm were the extraembryonic membranes are being patterning, the anterior-dorsal and ventral side of the embryo. (**F**) At later stages, *pri*-*Ame*-*mir*-*0002* RNA is detected in the neuroectoderm.

The novel miRNA *Amemir0002* is located within an intron of the *vesicular glutamate transporter 2*.*1* gene on chromosome 11 (Figure 3C). *PriAmemir0002* RNA was expressed throughout the ectoderm of the stage 4 embryo (Figure 3D), by late stage 5 RNA expression is no longer detected at the anterior-dorsal region of the embryo, or along the dorsal surface (Figure 3E), this includes areas where the extraembryonic membranes form [22]. Later *priAmemir0002* RNA expression was found throughout the neuroectoderm (Figure 3F). These expression patterns imply a possible role for mir-0002 in patterning the ectoderm and neuroectoderm during honeybee development. Many species-specific miRNAs are located in the introns of protein coding genes compared to those are proposed to be more ancient which tend to be intergenic [39]. Most are often transcribed from the host-gene promoter, although some intronic miRNAs have their own promoter [40,41]. Further work is required to determine if mir-0002 expression is driven from the host-gene promoter or if it has its own promoter region.

**Figure 4:**
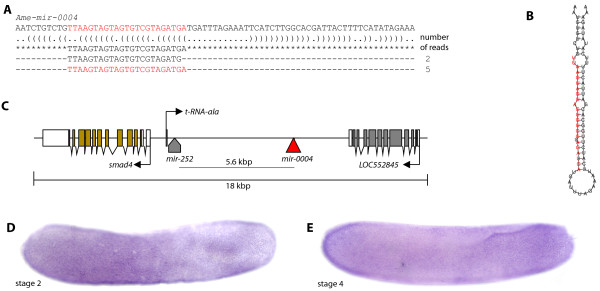
**Expression and sequence of novel Ame-mir-0004. **The embryo is orientated anterior to the left, dorsal side up. (**A**) Sequence of the predicted mir-0004 precursor with the sequence reads aligned below and corresponding read counts. Bracket-notation of secondary structure is shown. Highlighted in red is the sequence with the highest read counts. (**B**) The predicted mir-0004 hairpin structure, with the most abundant read sequence highlighted in red which is likely to correspond to the mature miRNA. (**C**) Intergenic location of the *pre*-*mir*-*0004* on chromosome 15. (**D**) Expression of *pri*-*Ame*-*mir*-*0004* during early embryogenesis. Nuclear expression is detected in the ectoderm at both the anterior and posterior poles. (**E**) Expression at stage 4 was detected throughout the embryo but enriched at each pole.

**Figure 5:**
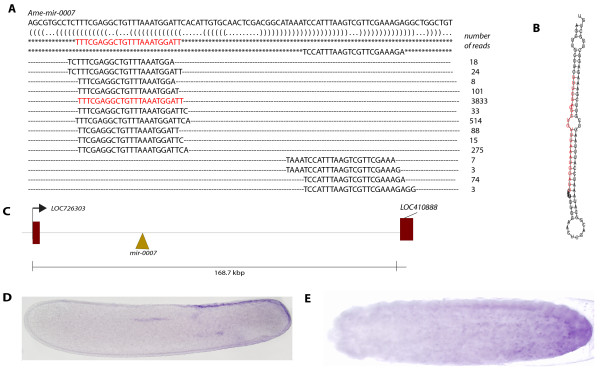
**Expression and sequence of novel Ame-mir-0007. **Embryos are positioned anterior to the left, dorsal side up. (**A**) Sequence of the mir-0007 precursor with aligned sequence reads and read counts. Highlighted in red is the sequence with the highest read counts. Bracket-notation of secondary structure is shown below the precursor. Mature miRNA and mirRNA* sequences are shown surrounded by asterisks. (**B**) The predicted mir-0007 hairpin structure, the most abundant read sequence highlighted in red. (**C**) Genomic location of the pre-mir-0007; mir-0007 is located between the predicted genes LOC726303 and LOC410888 on chromosome 2. (**D**) Expression during embryogenesis at stage 4. (**E**) Expression of pri-mir0007 at stage 9 is though the posterior of the extraembryonic membrane.

The novel miRNA mir-0004 is on chromosome 15, located between *smad4* and a hypothetical gene, and about 5.6 kbp from *mir*-*252* (Figure 4C). Expression of *pri*-*Ame*-*mir0004* RNA was detected in ectoderm cell nuclei at the anterior and posterior half of the embryo, but weaker within the central region of the embryo (Figure 4D). Embryo staining produced high background, probably as a result of longer staining times required to visualize the RNA expression; read count from the small RNA libraries indicated mir-0004 was present at low levels.

micr-0007 is located in the intergenic space between two predicted genes (Figure 5A). *Primir0007* RNA was detected in the dorsal-posterior ectoderm (Figure 5D), a part of the embryo where the amnion arises [22]. By stage 9, *primir0007* RNA was detected in the posterior half of the amnion (Figure 5E).

**Figure 6:**
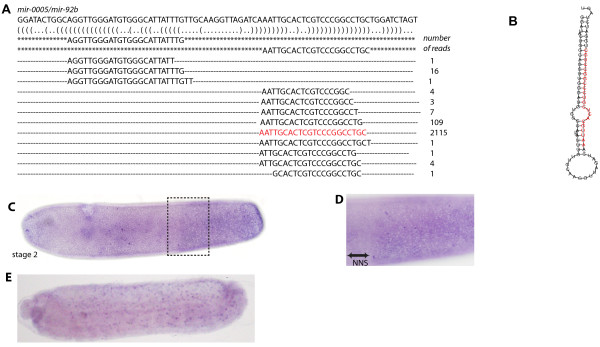
**Sequence and expression of Ame-mir-0005/mir-92b. **(**A**) Sequence of the novel mir-0005 precursor, with the sequence reads aligned below and associated read counts. Mature miRNA and miRNA* sequences are shown surrounded by asterisks. Bracket-notation of secondary structure is shown and highlighted in red is the sequence with the highest read count. (**B**) The predicted pre-mir-0005 hairpin structure, with the most abundant read sequence highlighted in red. (**C**-**F**) Embryonic expression during honeybee embryogenesis of *pri*-*Ame*-*mir*-*0005*. (**C**) Lateral view of a stage 4 embryo. *Pri*-*Ame*-*mir*-*0005* RNA expression was strongest in the posterior third of the embryo. (**D**) Higher magnification view of the boxed region in (**C**) showing nuclear dot staining but not in the adjacent region where only background staining was detected (no nuclear staining (NNS)). (**E**) Ventral view of a stage 9 embryo revealing staining in the amnion region of the embryo.

**Figure 7:**
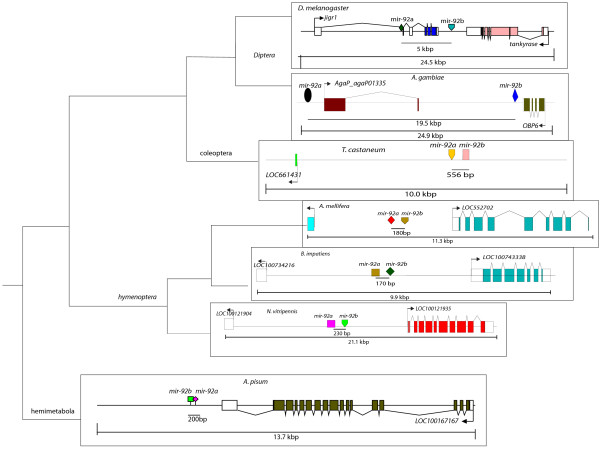
**Genomic location of *pre*-*mir*-*92a *and *pre*-*mir*-*92b *in arthropods. ***Mir*-*92a* and *b* are intragenic positioned closely in hemimetabola, hymenoptera and coleopteran genomes and are predicted be expressed as part of the same pri-RNA transcript. However in Diptera, *mir92a* and *b* are now separated by considerable distances and may no longer be part of the same polycistronic transcript.

Mir-0005 was detected with high numbers of reads (1642 and 2263 reads per sample) in our small RNA libraries. Analysis of the genomic location and mir-0005 mature sequence indicates that this is likely to be a paralogue of mir-92b (Figure 6A). Previously two *mir92b1* genes had been identified in the honeybee genome (mirBASE) but these are located on different chromosomes to that of mir-92a (Additional file 5: Figure S7), whereas mir-0005 is located next to *mir92a* and had not been reported previously. We examined the conservation of the *mir92a* -*92b* cluster in arthropods (Figure 7). In aphid, *Apis*, *Tribolium*, *Nasonia* and *Bombus*, *mir92a* and *mir92b* are clustered within 200 bp of each other in an intron. In Diptera (*Drosophila*, *Aedes* and *Anopholes*), they are no longer linked and are now separated by 5 to 50 kbp of DNA. This is similar to previously studies that indicate that miRNA clusters are conserved in most insects but become fragmented in *Diptera* lineage [18]. We found that Mir-92a was present at an almost ten-fold lower read count in our small RNA libraries compared to mir-0005/mir092b, indicating they are produced differentially despite being close enough to be processed from the same primary transcript (Figure 7 and Additional file 5: Figure S7). The pri-mir-0005 probe used to detect expression of RNA would detect a primary transcript that includes both mir-92a and mir-0005. Nuclear staining for *primir0005* RNA in honeybee was found to be present at the posterior ectoderm of stage 4 embryos (Figure 6C and D). Later, at stage 9, *primir0005* RNA expression was detected in the amnion (Figure 6F).

#### Knockdown of Dicer during early embryogenesis

**Figure 8:**
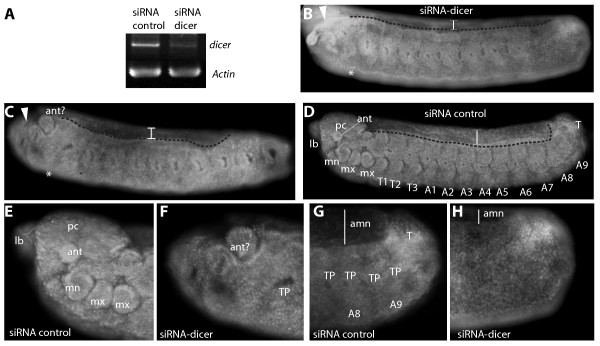
**Effect of Dicer knockdown on honeybee embryo development. **(**A**) Semi-quantitative PCR validation of reduction in *Am*-*dicer* mRNA levels in siRNA treated embryos compared to controls. (**B**-**C**) Examples of stage 9 (48 hour) embryos following siRNA targeted knockdown of Am-dicer. Head appendages are missing (asterisk), as are the procephalic lobes (arrowhead). The amnion covering the yolk is reduced in siRNA treated embryos (line, compare to control (D)). (**D**) Stage 9 embryos injected with control siRNA. (**E**) Anterior region of a stage 9 embryo injected with a non-targeted siRNA. (**F**) Closer view of the anterior of a siRNA-dicer injected embryo revealing loss of anterior appendages. (**G**) The posterior end of a control injected embryo. (**H**) The posterior terminal region of an siRNA dicer treated embryo, showing a reduced amnion (line) and loss of tracheal pits. Abbreviations: labrum (lb), mandible (mn), maxillae (mx), thorax segments (T1-3), abdominal segments (A1-9), terminal segment (T), antenna (ant), procephalic lobe (pc), tracheal pit (TP), amnion (amn).

To determine if miRNAs expressed during embryogenesis do indeed play a role in honeybee development, we knocked down dicer expression using siRNAs against *Amdicer* (Figure 7A). Dicer is a ribonuclease required for the production of mature miRNAs from the pre-miRNA precursor, thus by reducing levels of Dicer in the early embryo, we expect to target the processing of pre-miRNA precursors and thus decrease miRNA synthesis. Freshly laid honeybee embryos (approximately 400 each) were injected with siRNAs against *Amdicer*, or a control siRNA (Figure 8; Additional file 6). This strategy is likely to have resulted in knockdown of the zygotic production of small RNAs. Following injection, embryos were incubated for 48 hours and fixed and staining with DAPI to assess morphology. Approximately 20% survived through to stage 9, in line with previous survival ratios from similar procedures due to the nature of the injection procedure [22,23]. Posterior and anterior regions of the embryos were malformed with loss of terminal patterning (Figure 8B, C, F and H). In some embryos there was a significant reduction in the amnion that normally covers the yolk sac, and the dorsal regions of the embryo extend further towards the ventral side of the embryo (Figure 8B and H). This phenotype is very similar to that of *Amzen* RNAi knockdown [22], suggesting problems with dorso-ventral patterning. Abdominal tracheal pits were present but segments appeared to be fused together, indicating a defect in anterior-posterior patterning (Figure 8B and C).

## Conclusions

We examined the miRNA content of early honeybee embryos by deep sequencing followed by determination of the expression patterns of eight of these miRNAs. Consistent with both miRNA expression patterns and target prediction, Dicer siRNA knockdown embryos had defects in extraembryonic membrane formation, anterior-posterior and dorso-ventral patterning, suggesting that miRNAs may have functions in regulating these patterning pathways or their gene target(s) have roles in multiple pathways.

Many miRNAs that are expressed during embryo development are deeply conserved throughout metazoans indicating a more ancient origin. However, several are unique to the arthropod group, and some restricted just to the *Apis* lineage. Given the developmental expression of these miRNAs, they may have taken on roles particular to the development of the honeybee embryo. Previous studies have hypothesised that miRNAs are continuously added to the metazoan genomes, are stabilized once added, and are rarely lost [27,42,43].

Interestingly, some of the highly conserved miRNAs identified in our study had very different expression patterns in the honeybee to those documented in other animals. *Mir1* expression differed between *Apis* and other animals, which may result from loss of a regulatory element or binding sites for cis-regulatory proteins [44]. Early embryonic expression of *mir10* differed between *Drosophila* and *Apis*, but later expression was similar and is consistent with regulation by separate cis-regulatory elements (one controlling early expression, one controlling later expression) as suggested previously for *Drosophila* mir-10 [8]. This suggests that earlier expression regulatory elements are evolving more rapidly. A similar pattern of more labile expression in early development has previously observed for protein-coding genes [24,45-47]. These changes or shifts in miRNA expression imply the regulatory regions controlling miRNA expression are also rapidly evolving. This indicates the importance that changes to gene-regulatory sequences contribute to the evolution of developmental pathways extends to also changes associated with regulatory elements that control miRNA genes.

## Methods

### Sample collection and preparation

A queen honeybee was caged with an empty area of an Eziqueen queen rearing frame and placed back into the hive. After 5 hours the frame was removed and the queen released back into the hive. The eggs were removed and while still attached to the black strips of the Eziqueen frame incubated for 24 hours. Eggs were collected and total RNA extracted using TRIzol (Life Technologies). Total RNA concentration and purity determined was using a Nanodrop spectrometer (Thermo Scientific). 10 μg of purified total RNA was sent to Beijing Genomics Institute (BGI) for sequencing on an Illumina HiSeq 2000 sequencer. Low quality reads, reads without the adaptors, reads with polyA sequences and reads without the insert tag where removed. Also discarded were any tRNA, rRNA, snRNA and snoRNA sequences. Sequence reads were mapped to the genome using the programme SOAP [44]. Small RNA tags were aligned to known miRNA *Apis mellifera* precursors (miRBASE). The two small RNA libraries shared 98.06% of sequences. Analyses of the length distribution of cleaned reads showed enrichment of small RNAs from 22 to 31 nucleotides (Additional file 7: Figure S1); we would expect about a length of 22nt for miRNAs. Examination of the first base of the 22 nucleotide sequences revealed most show a first base bias to uridine as predicted from previous deep sequencing miRNA studies (Additional file 8: Figure S2).

### Amplication of pri-miRNA fragments

Oligonucleotide primers were designed to amplify 500-800 bp regions using genomic template. In each amplicon, the precursor miRNA resides in the centre of the sequence. PCR fragments were cloned into the vector pBluescript II KS (+/-) and sequenced. Cloned fragments were used to produce RNA sense and antisense *in situ* hybridisation probes. Oligonucleotide primer pairs were as follows: mir-10 5^′^ACAAATGGACGACGAAGAGG3^′^ and 5^′^GCGGCACGTACGTTACTTTA3^′^, mir-1 5^′^GCCACGTACGTTCGAAAACT3^′^ and 5^′^TTCGCAAGACGGATACATCA3^′^, mir-184 5^′^ GCCTCGGGTTTCGAGGCGTT3^′^ and 5^′^ AGGAGAAGGGAAGAATGTGCAGAGA3^′^, mir-9a 5^′^CCGATTTCTCCGTCTTTTCTG3^′^ and 5^′^CCGATTTCTCCGTCTTTTCTG3^′^, mir-0002 TGTACGGGCAGTACTGGG and TCTTGATGATGCGTCTTG, mir-0004 5^′^CAACGATGCGTTTCGACTTA3^′^ and 5^′^GTACCCACGAGTCGTCAC3^′^, mir-0005 5^′^TCGATATTCGAAACGCAACA3^′^ and 5^′^TGGATTTGAATTCGTGTATGAAA3^′^, mir-0007 5^′^ACGAGGATACACGGATGGAC3^′^ and CAATTCACTTCCTTTTCACCTCA3^′^.

### *In situ* hybridization on honeybee embryos

Performed as per Osborne [48] with the following modifications. Incubations with pri-miRNA anti-sense and sense probes were carried out at 60°C with rotation for 48 hours before post-hybridisation wash steps to remove excess probe. Embryos were incubated overnight at 4°C with anti-dioxygenin-alkaline phosophatase antibody with rotation before post-antibody wash steps and colour reaction.

### Dicer siRNA knockdown

Two siRNAs were designed against Am-dicer; GGACGAAGAGUUAGAGUUAUU and UGAAACAGCUAGUGAUAUAUU. The two siRNAs were injected together at a final concentration of 5 μg/ml into freshly laid eggs attached to plastic strips from an Eziqueen frame [49]. As a control, a non-targeting siRNA (D-001810-01-05, Dharmacon) was also injected at the same concentration. Following injection, embryos were placed in a humidified incubator at 35°C for 48 hours. After incubation they were fixed with heptane/formaldehyde in PBS overnight, rocking at room temperature. After fixation, embryos were washed with PBS and stained with DAPI before visualization on an Olympus BX61 microscope with a DP71 camera. Embryos were staged as per DuPraw [19].

## Competing interests

The authors declare they have no competing interests.

## Authors’ contributions

MJW performed most of the *in situ* hybridisations, siRNA experiments and designed the project and wrote the publication. LZ cloned and synthesized the *in situ* probes, performed some of the honeybee *in situ* hybridisations and was involved in the writing of the manuscript. PKD discussed the data and took part in writing the manuscript. All authors read and approved the final manuscript.

## Supplementary Material

Additional file 1 **Figure S3. **(**A**) Pseudocoloured image showing DAPI (blue) and *Am*-*eve* RNA staining in red. *Am*-*eve* RNA is detected in the cytoplasm of embryonic cells. (**B**) Pseudocoloured image of *pri*-*mir*-*0002* RNA staining (red) overlaid with DAPI (blue) staining. *Pri*-*mir*-*0002* RNA is detected in the nucleus of embryonic cells. Click here for file

Additional file 2 **Figure S4. Genome location and read count for ***Ame*-*mir*-*10.* Genome location of *Ame*-*mir*-*10* in the *Hox* complex, between *deformed* (*dfd*) and *sex*-*combed reduced* (*Scr*). Click here for file

Additional file 3 **Figure S5. Clusterdraw analysis of the upstream regions of *Dme*-*mir*-*1*and*Ame*-*mir*-*1. ***Cluster of twi binding sites using the clusterdraw programme [50] with background model either site at *D*. *melanogaster* (A) or *A*. *mellifera* (B). This programme has successfully identified cis-regulatory elements in *Apis* and *Drosophila* previously [22,50-52]. *P* values cut off on the Y-axis and position in the sequence along the x-axis. Click here for file

Additional file 4 **Figure S6. Alignment of mir-0008 and mir-0005/mir-92b pre-miRNAs. Abbreviations: ***Apis mellifera*, *Bombus impatiens*, *Atta cephalotes*, *Nasonia vitripennis*, *Drosophila melanogaster*. Boxed are the mature miRNA sequences. Click here for file

Additional file 5 **Figure S7. Alignment of sequence reads to ***Apis* mir-92a, mir-92b-1 and mir-0005/mir-92b pre-miRNAs. Click here for file

Additional file 6 Table S1. Phenotype of surviving larvae (at 72 hours) following siRNA injections. Click here for file

Additional file 7 Figure S1. Length distribution in both samples of clean small RNA reads. Click here for file

Additional file 8 Figure S2. miRNA nucleotide bias at each position. Click here for file
