# Supplementary figures and images for: Deep sequencing and expression of microRNAs from early honeybee (Apis mellifera) embryos reveals a role in regulating early embryonic patterning
Source: BMC Evol Biol. 2012 Nov 2;12:211. doi: 10.1186/1471-2148-12-211 (PMC3562263; doi:10.1186/1471-2148-12-211)

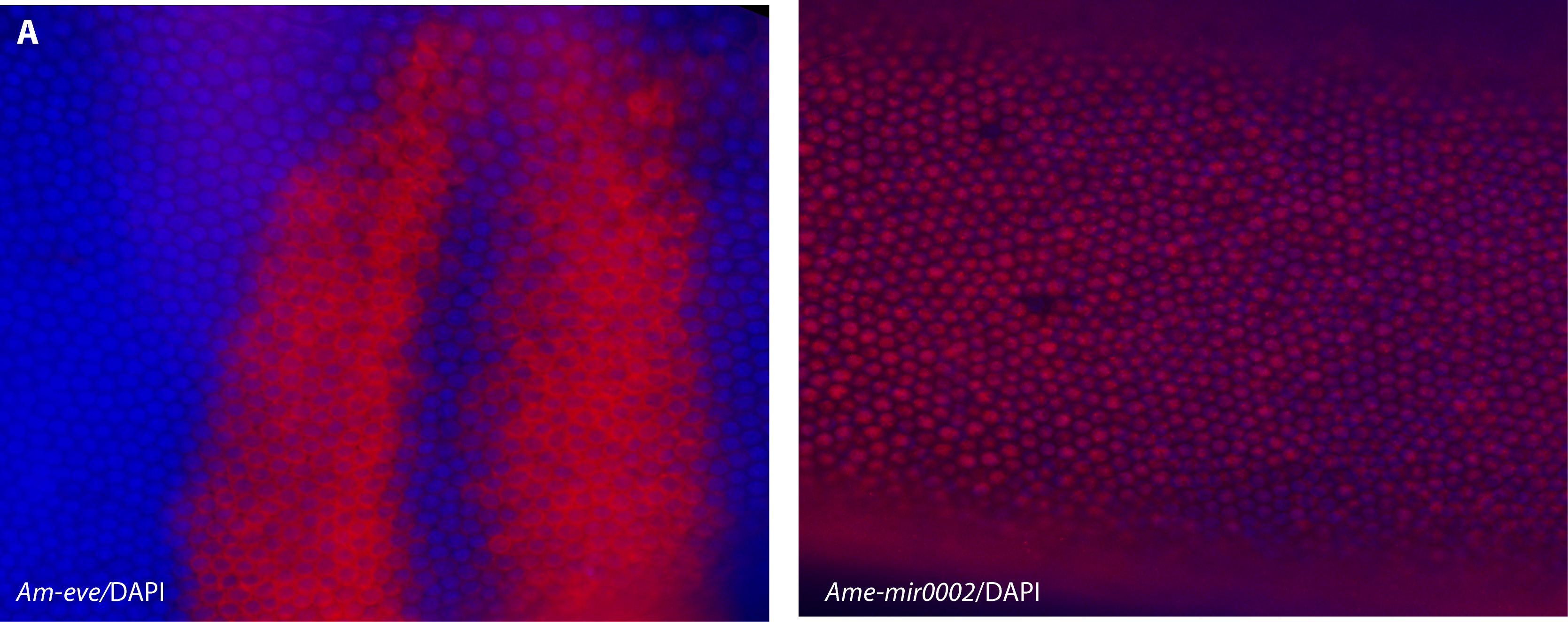

Supplement: Additional file 1 — Figure S3. (A) Pseudocoloured image showing DAPI (blue) and Am-eve RNA staining in red. Am-eve RNA is detected in the cytoplasm of embryonic cells. (B) Pseudocoloured image of pri-mir-0002 RNA staining (red) overlaid with DAPI (blue) staining. Pri-mir-0002 RNA is detected in the nucleus of embryonic cells. [file 1471-2148-12-211-S1.tiff]

Group16.4

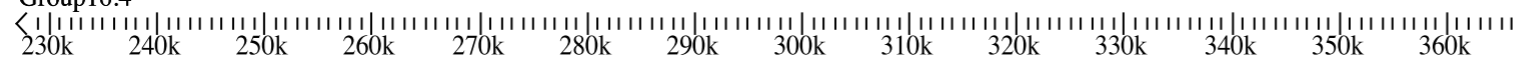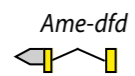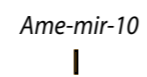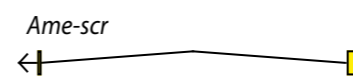

Supplement: Additional file 2 — Figure S4. Genome location and read count for Ame-mir-10. Genome location of Ame-mir-10 in the Hox complex, between deformed (dfd) and sex-combed reduced (Scr). [file 1471-2148-12-211-S2.pdf]

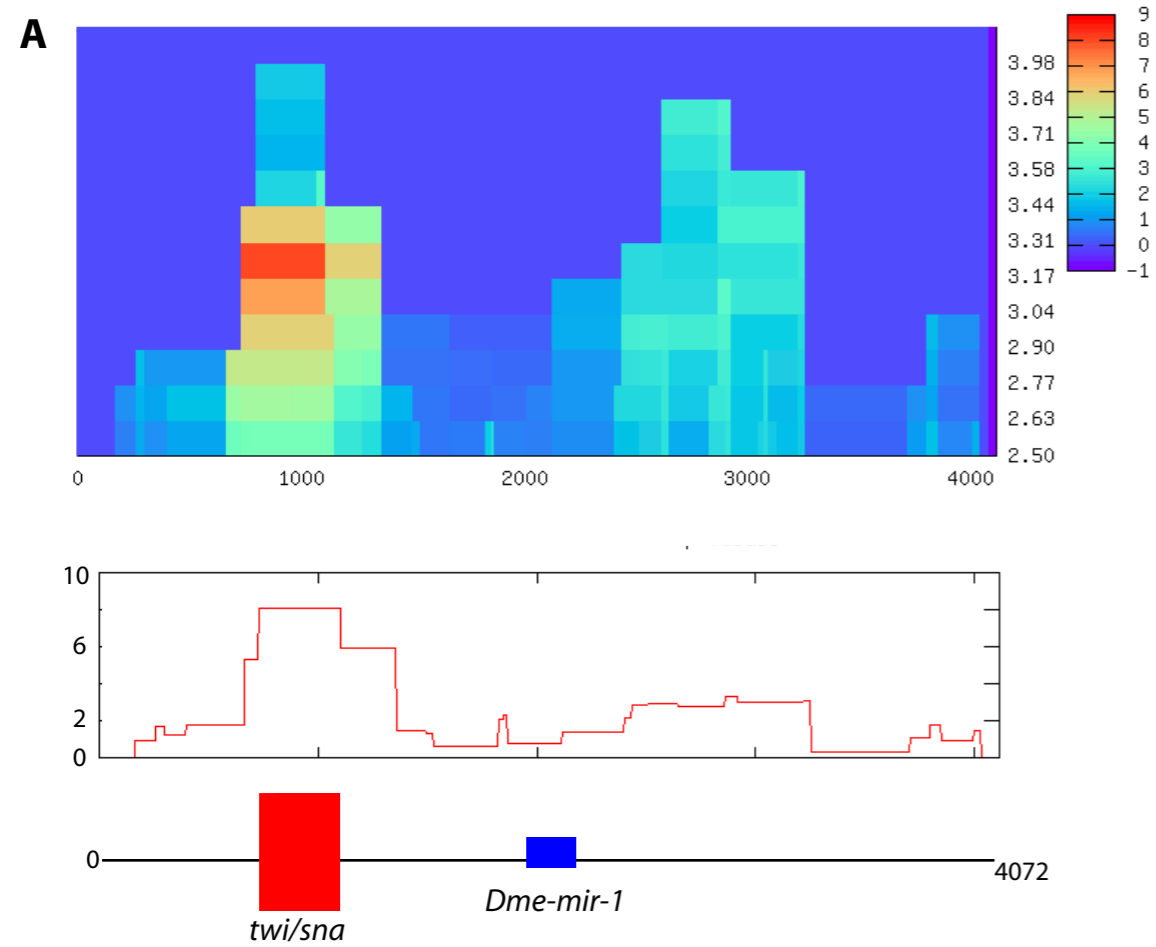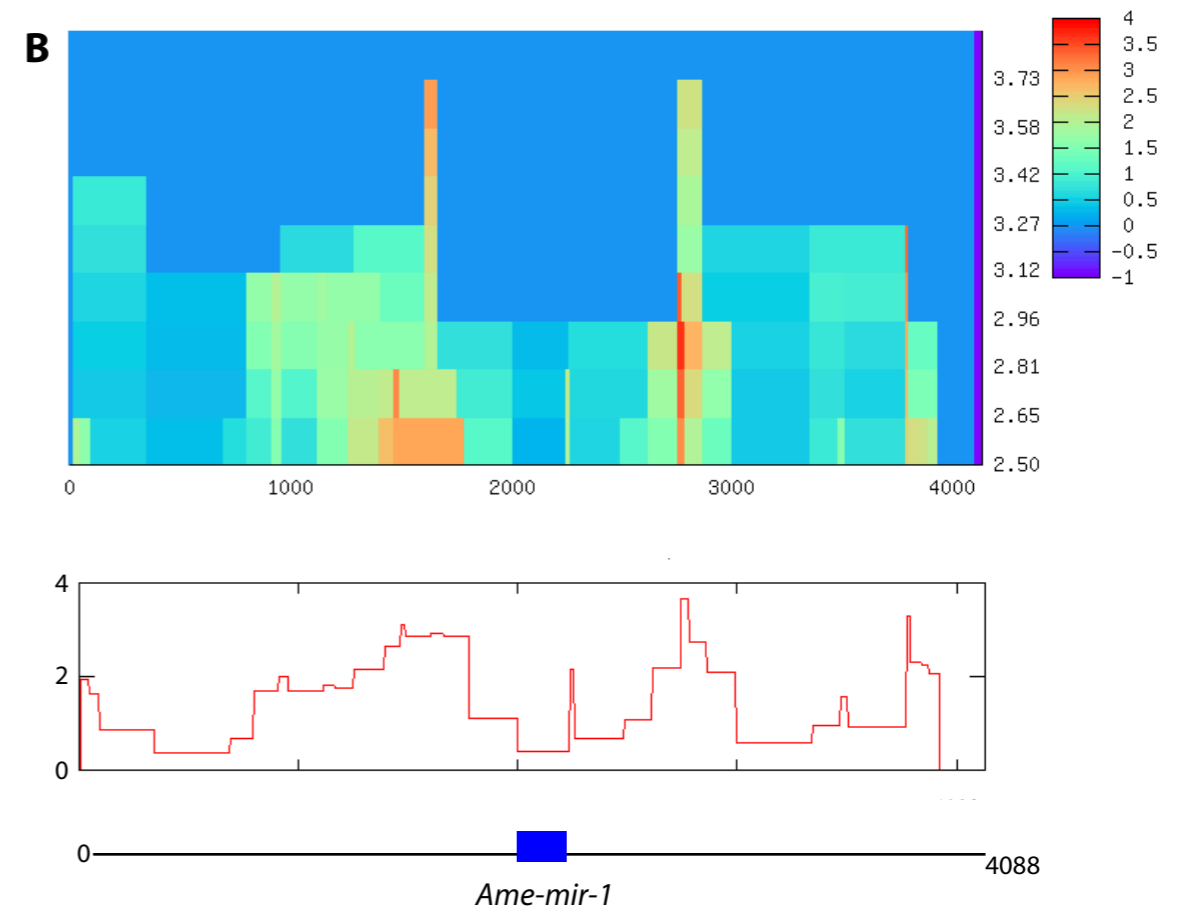

Supplement: Additional file 3 — Figure S5. Clusterdraw analysis of the upstream regions of Dme-mir-1andAme-mir-1. Cluster of twi binding sites using the clusterdraw programme [50] with background model either site at D. melanogaster (A) or A. mellifera (B). This programme has successfully identified cis-regulatory elements in Apis and Drosophila previously [22,50-52]. P values cut off on the Y-axis and position in the sequence along the x-axis. [file 1471-2148-12-211-S3.pdf]

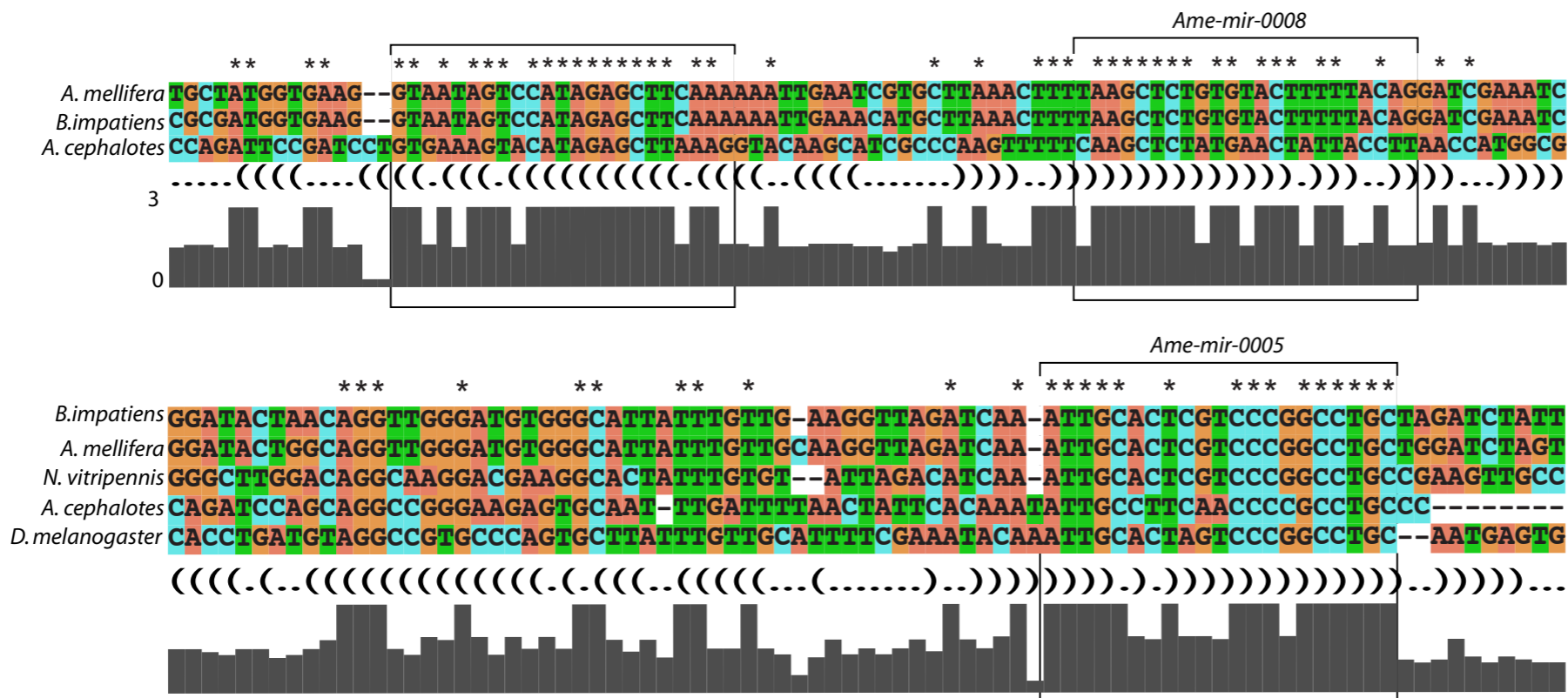

Supplement: Additional file 4 — Figure S6. Alignment of mir-0008 and mir-0005/mir-92b pre-miRNAs. Abbreviations: Apis mellifera, Bombus impatiens, Atta cephalotes, Nasonia vitripennis, Drosophila melanogaster. Boxed are the mature miRNA sequences. [file 1471-2148-12-211-S4.pdf]

# Length Distribution - sample 1

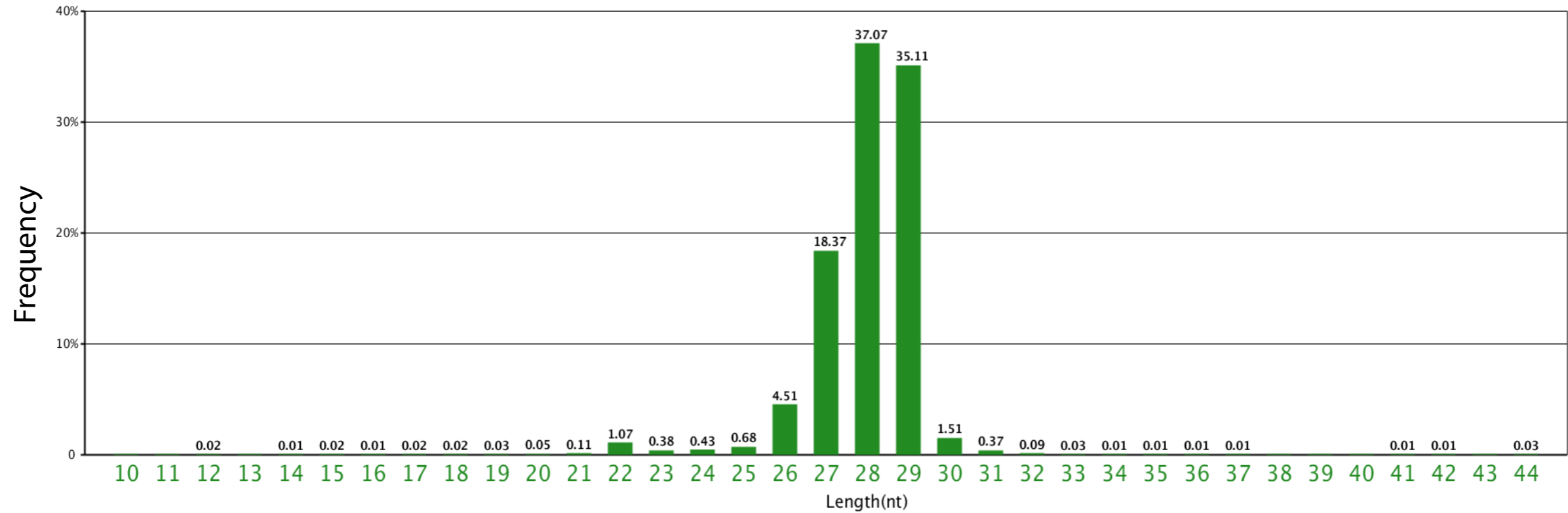

# Length Distribution - sample 2

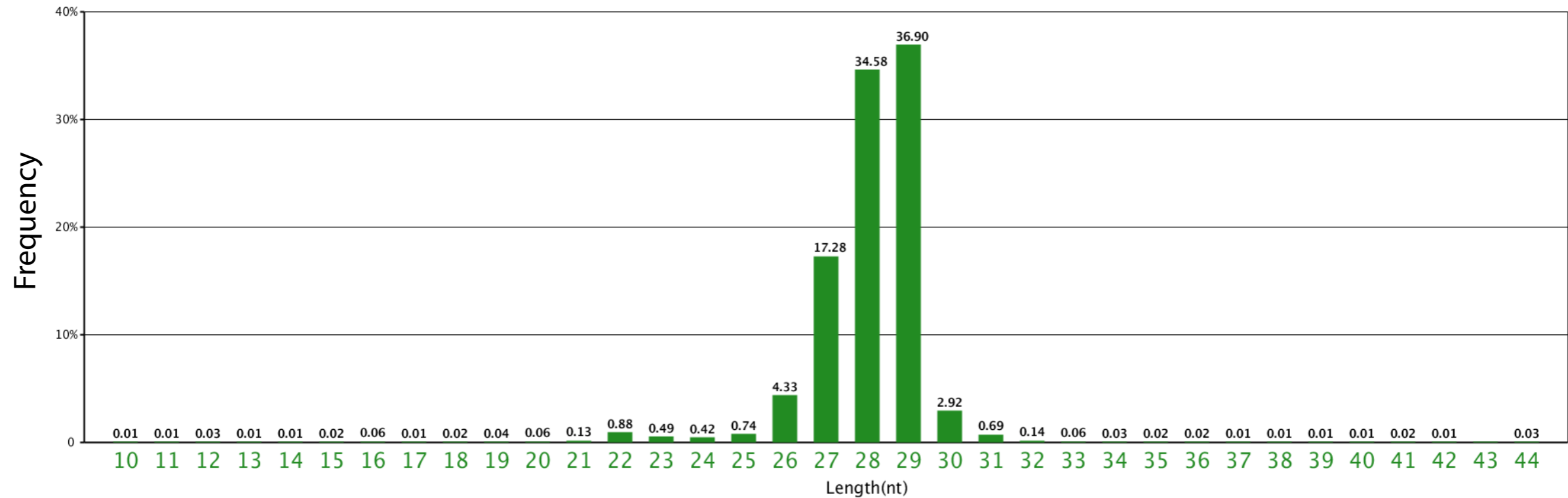

Supplement: Additional file 7 — Figure S1. Length distribution in both samples of clean small RNA reads. [file 1471-2148-12-211-S7.pdf]

miRNA nucleotide bias at each position

sample 1

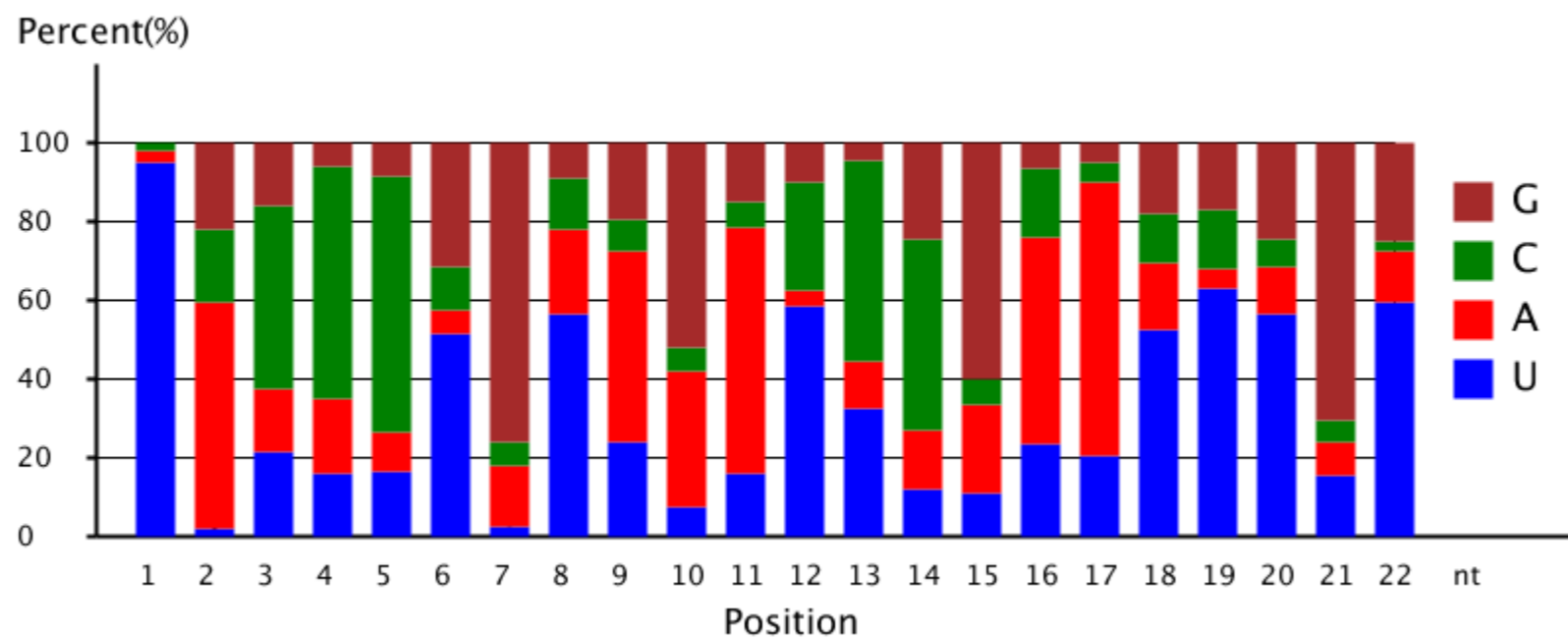

miRNA nucleotide bias at each position

sample 2

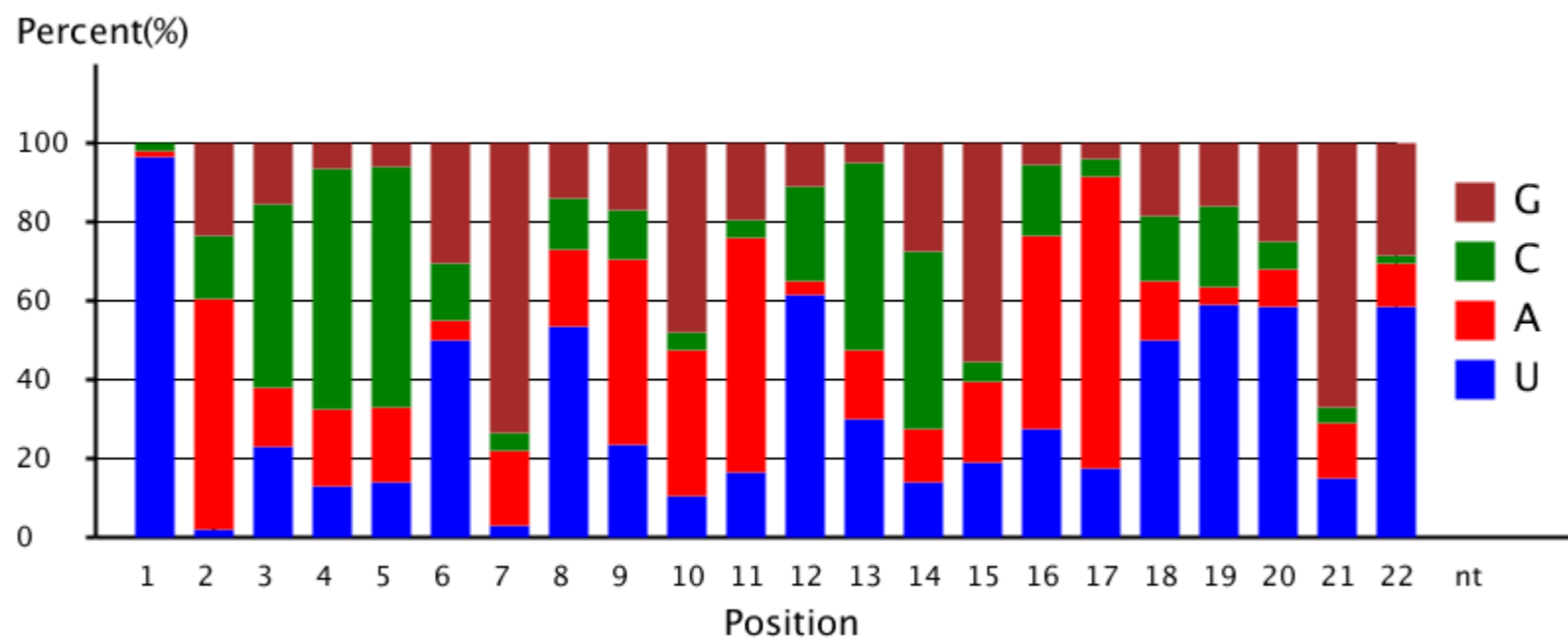

Supplement: Additional file 8 — Figure S2. miRNA nucleotide bias at each position. [file 1471-2148-12-211-S8.pdf]
