# Supplementary material for: Deep sequencing and expression of microRNAs from early honeybee (Apis mellifera) embryos reveals a role in regulating early embryonic patterning
Source: BMC Evol Biol. 2012 Nov 2;12:211. doi: 10.1186/1471-2148-12-211 (PMC3562263; doi:10.1186/1471-2148-12-211)
Supplement: Additional file 5 — Figure S7. Alignment of sequence reads to Apis mir-92a, mir-92b-1 and mir-0005/mir-92b pre-miRNAs. [file 1471-2148-12-211-S5.pdf]

```
mir-92a Chromosome 15
TTATTTTGCATAGAAGATAGGCCGAGATTTGTGACAATGTTTCGTGATGATGTAATCTTCAATATTGCACTTGTCCCGGCCTATCGGAATGCATTATATT ame-mir-92a 100
.(((..(((((....(((((((((.(((..(((.(((((((....(((......)))...))))))))))..))).)))))))))...)))))..)))..
***************************************************************ATTGCACTTGTCCCGGCCTAT**************** ame-miR-92a 21
.................TAGGCCGAGATTTGTGACAATGTT........................................................... t0357335 24 1
..................AGGCCGAGATTTGTGACAATGTT........................................................... t0026282 23 13
..................AGGCCGAGATTTGTGACAATG............................................................. t0213235 21 1
..............................................................TATTGCACTTGTCCCGGCCTAT................ t0003746 22 210
..............................................................TATTGCACTTGTCCCGGCCTA................. t0073596 21 3
..............................................................TATTGCACTTGTCCCGGC.................... t0191671 18 1
mir-92b-1  Chromsome 9
CACGAAGCGTATTCAATTAGGTCAGGACTAATGCAAATATGTAGTGTCGACATCAAATTGCACCCGTCCCGGCCTGATTGAGTGCGCGTAAATC ame-mir-92b-1 94
......(((((((((((((((((.((((...(((((..((((.......))))....)))))...)))).))))))))))))))))).......
*******************************************************AATTGCACCCGTCCCGGCCTGA***************** ame-miR-92b 22
..................AGGTCAGGACTAATGCAAATATG..................................................... t0036818 23 8
..................AGGTCAGGACTAATGCAAATATGTAGTG................................................ t0311239 28 1
.......................................................AATTGCACCCGTCCCGGCCTGA................. t0004923 22 145
.......................................................AATTGCACCCGTCCCGGCCTG.................. t0093334 21 2
.......................................................AATTGCACCCGTCCCGGCC.................... t0290940 19 1
........................................................ATTGCACCCGTCCCGGCCTGA................. t0378943 21 1

mir-0005 (now mir-92b)  Chromosome 15
GGATACTGGCAGGTTGGGATGTGGGCATTATTTGTTGCAAGGTTAGATCAAATTGCACTCGTCCCGGCCTGCTGGATCTAGT m0005 2263
((((.(..(((((((((((((.(.(((..(((((...(.......)..)))))))).).)))))))))))))..)))))...
**********AGGTTGGGATGTGGGCATTATTTG************************************************ m0005-5p 18
**************************************************AATTGCACTCGTCCCGGCCTGC********** m0005-3p 2245
----------AGGTTGGGATGTGGGCATTATT-------------------------------------------------- t0157426 1
----------AGGTTGGGATGTGGGCATTATTTG------------------------------------------------ t0023023 16
----------AGGTTGGGATGTGGGCATTATTTGTT---------------------------------------------- t0154308 1
--------------------------------------------------AATTGCACTCGTCCCGGC-------------- t0057586 4
--------------------------------------------------AATTGCACTCGTCCCGGCC------------- t0076834 3
--------------------------------------------------AATTGCACTCGTCCCGGCCT------------ t0039423 7
--------------------------------------------------AATTGCACTCGTCCCGGCCTG----------- t0006032 109
--------------------------------------------------AATTGCACTCGTCCCGGCCTGC---------- t0000602 2115
--------------------------------------------------AATTGCACTCGTCCCGGCCTGCT--------- t0252919 1
---------------------------------------------------ATTGCACTCGTCCCGGCCTG----------- t0364663 1
---------------------------------------------------ATTGCACTCGTCCCGGCCTGC---------- t0063068 4
------------------------------------------------------GCACTCGTCCCGGCCTGC---------- t0339302 1
```
