# Supplementary material for: Deep sequencing and expression of microRNAs from early honeybee (Apis mellifera) embryos reveals a role in regulating early embryonic patterning
Source: BMC Evol Biol. 2012 Nov 2;12:211. doi: 10.1186/1471-2148-12-211 (PMC3562263; doi:10.1186/1471-2148-12-211)
Supplement: Additional file 6 — Table S1. Phenotype of surviving larvae (at 72 hours) following siRNA injections. [file 1471-2148-12-211-S6.doc]

**Supplementary Table 1** Phenotype of surviving larvae (at 72 hours) following siRNA injections.

| **Injected siRNA** | **Defects in D/V patterning and anterior/posterior terminal poles** | **WT embryos** |
| --- | --- | --- |
| **siRNA against Dicer** | 35 | 20 |
| **siRNA control** | - | 63 |
